# Panton-Valentine Leukocidin–Positive MRSA, Shanghai

**DOI:** 10.3201/eid1604.081324

**Published:** 2010-04

**Authors:** Li-Zhong Han, Pak-Leung Ho, Yu-Xing Ni, Hong Zhang, Yan-Qun Jiang, Hai-Qing Chu, Yang Sun, Yi-Bo Zhang

**Affiliations:** Ruijin Hospital, Shanghai, People’s Republic of China (L.-Z. Han, Y.-X. Ni, Y.-B. Zhang); University of Hong Kong, Hong Kong Special Administrative Region, PRC (P.-L. Ho); Shanghai Children’s Hospital, Shanghai (H. Zhang); Sixth People’s Hospital of Shanghai, Shanghai (Y.-Q. Jiang); Shanghai Pulmonary Hospital, Shanghai (H.-Q. Chu); Minhang Central Hospital, Shanghai (Y. Sun)

**Keywords:** Staphylococcus aureus, MRSA, staphylococci, bacteria, pneumonia, Panton-Valentine leukocidin, multilocus sequence typing, molecular epidemiology, China, letter

**To the Editor**: The development of methicillin resistance in community strains of *Staphylococcus aureus* is a notable step in the evolution of this pathogen. Unlike their equivalents in the hospital environment, community-associated methicillin-resistant *S. aureus* (CA-MRSA) strains tend to cause infections in children and young adults who have few known healthcare risks (*1*). CA-MRSA strains usually possess the Panton-Valentine leukocidin (PVL) genes and staphylococcal cassette chromosome (SCC) *mec* type IV or V (*1*,*2*).

We studied 72 *S. aureus* isolates (49 MRSA and 23 methicillin-susceptible [MSSA]) by pulsed-field gel electrophoresis and by SCC*mec*, staphylococcal protein A (spa), and multilocus sequence typing (*1*,*3*). These isolates were recovered from clinical specimens (52 respiratory specimens, 9 wound, 4 urine, 2 blood, and 5 other body fluids) from 72 patients treated in 5 district hospitals in Shanghai, People’s Republic of China, during October 2005 through January 2007. The isolates were randomly chosen. In the hospitals, ≈1,000 *S. aureus* isolates were recovered annually during the time period of our study. The 5 hospitals are estimated to serve a population of 3.4 million, equivalent to one fourth of the total population in Shanghai. Hospital D is a children’s hospital. The other 4 hospitals (A, B, C, and E) have all the major clinical specialties, emergency departments, and outpatient clinics.

The isolates were identified as *S. aureus* by Gram stain, latex agglutination (Slide StaphPlus; bioMérieux, Marcy l’Etoile, France), and tube coagulase, mannitol, ornithine, and deoxyribonuclease reactions (*1*,*4*). Methicillin resistance in the isolates was detected by cefoxitin disc screening and confirmed by *mecA* PCR (*1*,*4*). For patients with PVL-positive MRSA, the computerized discharge records in the hospitals were retrospectively reviewed to ascertain demographic and clinical information. A MRSA case was considered to be community associated if it was isolated from an outpatient or within 2 days of a patient’s hospitalization. Exclusion criteria included a history of hospitalization for illness (except birth), surgery, or dialysis in the previous year or the presence of indwelling catheters or other medical devices (*1*). Conversely, healthcare-associated MRSA was defined by isolation >2 days after hospitalization or presence of any of the aforementioned healthcare risks.

PVL genes were detected in 9 (18.4%) of the 49 MRSA isolates (Table) and 4 (17.3%) of the 23 MSSA isolates. The 9 MRSA case-patients included 8 infants with pneumonia and 1 adult with prostatitis. Pulsed-field gel electrophoresis clustered 8 of the 9 PVL-positive MRSA isolates into 2 groups: 6 isolates as SH100 and 2 isolates as SH200. Strains of SH100 were *spa* type/MLST-SCC*mec* type t318/ST30-IV or t318/ST1114-V, and SH200 strains had t1376/ST88-V. Similar to the PVL-positive MRSA isolates, a limited number of *spa* types were found among the 40 PVL-negative MRSA isolates. These were t037/ST239-III (n = 19), t002/ST5-II (n = 14), t030/ST239-III (n = 5), t459/ST239-III (n = 1), and t1764/ST88-IV (n = 1).

**Table:** Epidemiologic and microbiologic characteristics for Panton-Valentine leukocidin–positive MRSA infections in 9 case-patients, Shanghai, People’s Republic of China, 2006*

Strain	Age/sex	Admission month	Onset	Diagnosis†	Resistance pattern‡	Resistance determinants§	ST§	SCC*mec*	*spa* type	PFGE
D8	1 mo/F	Feb	HA	Pneumonia	Chl, Ery, Gen, Mup	*aacA-aphD*, *ermC*, *ileS-2*	ST30	IV	t318	SH100
D9	1 mo/F	Feb	CA	Pneumonia	Gen, Mup	*aacA-aphD*, *ileS-2*	–	IV	t318	SH100
D13	1 mo/M	Mar	HA	Pneumonia	Gen	*aacA-aphD*	–	IV	t318	SH100
D14	3 mo/M	Mar	CA	Pneumonia	Gen	*aacA-aphD*	–	IV	t318	SH100
D16	1 mo/M	Mar	CA	Pneumonia	Gen, Mup	*aacA-aphD*, *ileS-2*	–	IV	t318	SH100
D12	3 mo/F	Mar	HA	Pneumonia	Ery	*ermC*	ST1114	V	t318	SH100
A18	29 y/M	Mar	HA	Prostatitis	None	–	ST30	IV	t3384	Singleton
D7	1 mo/F	Jan	CA	Pneumonia	Ery, Gen	*aacA-aphD*, *ermC*	ST88	V	t1376	SH200
D18	4 mo/M	Apr	CA	Pneumonia	Ery, Gen	*aacA-aphD*, *ermC*	–	V	t1376	SH200

*Strains are designated by hospital code and strain number. MRSA, methicillin-resistant *Staphylococcus aureus*; ST, sequence type; SCC, staphylococcal cassette chromosome; *spa*, staphylococcal protein A; PFGE, pulsed-field gel electrophoresis; HA, healthcare-associated; Chl, chloramphenicol; Ery, erythromycin; Gen, gentamicin, Mup, mupirocin; *acA-aphD*, aminoglycoside resistance gene encoding the bifunctional enzyme, 6'-aminoglycoside N-acetyltransferase/2”-aminoglycoside phosphotransferase; *ermC*, gene encoding macrolide-lincosamide-streptogramin B resistance; *ileS-2*, gene encoding high-level mupirocin resistance mediated by isoleucyl tRNA-synthetase; CA, community-associated. †According to the diagnosis given in the computerized record. In the 8 children, MRSA was isolated from sputum specimens. One child also had MRSA isolated from pleural fluid. Because limited information was provided in the computerized records, some of the isolations may represent respiratory colonization. The outcomes of the 8 children were unknown. ‡According to disk diffusion test (*1*,*5*). §Determined by PCR as described (*4*,*6*). ST30 (allelic profile 2–2-2–2-6–3-2) and ST114 (139–2-2–2-6–139–2) belonged to clonal complex 30.

In contrast, *spa* and sequence types (STs) among the 23 MSSA isolates were highly diverse. There were 20 *spa* types and 14 STs, giving a total of 20 distinct patterns. Three patterns (t091/ST7, t3388/ST630, t3389/ST15) had 2 isolates, and 17 patterns (t002/ST5, t1077/ST121, t127/ST1, t1376/ST88, t189/ST188, t2024/ST30, t2092/ST121, t2207/ST1206, t2471/ST25, t258/ST25, t3383/ST20, t3386/ST630, t377/ST630, t437/ST1205, t548/ST5, t701/ST6, t796/ST7) had 1 isolate only. The 4 PVL-positive MSSA isolates were t1376/ST88, t2471/ST25, t258/ST25, and t3383/ST20.

Mupirocin resistance rates among the PVL-positive and PVL-negative MRSA isolates were 33.3% (3/9) and 7.5% (3/40), respectively (p = 0.07). All MSSA isolates were susceptible to mupirocin.

It is notable that of the 9 PVL-positive MRSA isolates, 8 of them came from hospital D and all were from children 1–4 months of age. Others have noted that the epidemiology of MRSA differs for children and adults (*1*,*2*,*7**,**8*). Molecular typing showed that the PVL-positive CA-MRSA isolates were attributed to 2 clones with genotypes t318/ST30-IV (or t318/ST1114-V) and t1376/ST88-V. Detection of t318/ST30 strains in 4 patients with healthcare-associated infections suggested hospital transmission of this CA-MRSA clone, corroborating reports elsewhere (*9*). Worldwide, ST30 is a common CA-MRSA genetic lineage (*1*,*2*). Besides t318, strains related to the ST30 clone have been reported to be spa types t019, t021, and t1273 (*2*). ST88 PVL-positive MRSA is relatively less common but has been found in Wenzhou (People’s Republic of China), Bangladesh, Belgium, and Nigeria (*2*,*7**,**8**,**10*).

Because the number of isolates tested in this study is relatively small, no firm conclusion could be drawn on the prevalence of PVL-positive CA-MRSA among *S. aureus* isolates. Nonetheless, our findings agree with previous reports that the genotypes of MSSA isolates are more diverse than are those for PVL-positive and -negative MRSA isolates and that genotypes for some CA-MRSA strains are shared by a few of the MSSA strains (*1*).

## References

[1] Ho PL, Chuang SK, Choi YF, Lee RA, Lit AC, Ng TK, Community-associated methicillin-resistant and methicillin-sensitive *Staphylococcus aureus*: skin and soft tissue infections in Hong Kong. Diagn Microbiol Infect Dis. 2008;61:245–50. 10.1016/j.diagmicrobio.2007.12.015 18272316

[2] Tristan A, Bes M, Meugnier H, Lina G, Bozdogan B, Courvalin P, Global distribution of Panton-Valentine leukocidin-positive methicillin-resistant *Staphylococcus aureus*, 2006. Emerg Infect Dis. 2007;13:594–600. 10.3201/eid1304.061316 17553275 PMC2725977

[3] Ho PL, Lai EL, Chow KH, Chow LS, Yuen KY, Yung RW. Molecular epidemiology of methicillin-resistant *Staphylococcus aureus* in residential care homes for the elderly in Hong Kong. Diagn Microbiol Infect Dis. 2008;61:135–42. 10.1016/j.diagmicrobio.2007.12.017 18272314

[4] Ho PL, Wang TK, Ching P, Mak GC, Lai E, Yam WC, Epidemiology and genetic diversity of methicillin-resistant *Staphylococcus aureus* strains in residential care homes for elderly persons in Hong Kong. Infect Control Hosp Epidemiol. 2007;28:671–8. 10.1086/517951 17520539

[5] Finlay JE, Miller LA, Poupard JA. Interpretive criteria for testing susceptibility of staphylococci to mupirocin. Antimicrob Agents Chemother. 1997;41:1137–9. 9145883 10.1128/aac.41.5.1137 PMC163864

[6] Perez-Roth E, Claverie-Martin F, Villar J, Mendez-Alvarez S. Multiplex PCR for simultaneous identification of *Staphylococcus aureus* and detection of methicillin and mupirocin resistance. J Clin Microbiol. 2001;39:4037–41. 10.1128/JCM.39.11.4037-4041.2001 11682527 PMC88484

[7] Denis O, Deplano A, De Beenhouwer H, Hallin M, Huysmans G, Garrino MG, Polyclonal emergence and importation of community-acquired methicillin-resistant *Staphylococcus aureus* strains harbouring Panton-Valentine leucocidin genes in Belgium. J Antimicrob Chemother. 2005;56:1103–6. 10.1093/jac/dki379 16223937

[8] Yu F, Chen Z, Liu C, Zhang X, Lin X, Chi S, Prevalence of *Staphylococcus aureus* carrying Panton-Valentine leukocidin genes among isolates from hospitalised patients in China. Clin Microbiol Infect. 2008;14:381–4. 10.1111/j.1469-0691.2007.01927.x 18190580

[9] Zaoutis TE, Toltzis P, Chu J, Abrams T, Dul M, Kim J, Clinical and molecular epidemiology of community-acquired methicillin-resistant *Staphylococcus aureus* infections among children with risk factors for health care–associated infection: 2001–2003. Pediatr Infect Dis J. 2006;25:343–8. 10.1097/01.inf.0000207403.67197.cc 16567987

[10] Ghebremedhin B, Olugbosi MO, Raji AM, Layer F, Bakare RA, Konig B, Emergence of a community-associated methicillin-resistant *Staphylococcus aureus* strain with a unique resistance profile in Southwest Nigeria. J Clin Microbiol. 2009;47:2975–80. 10.1128/JCM.00648-09 19571020 PMC2738091

